# Serial mutational tracking in surgically resected locally advanced colorectal cancer with neoadjuvant chemotherapy

**DOI:** 10.1038/s41416-018-0208-5

**Published:** 2018-08-03

**Authors:** Keishi Sugimachi, Shotaro Sakimura, Shotaro Kuramitsu, Hidenari Hirata, Atsushi Niida, Tomohiro Iguchi, Hidetoshi Eguchi, Takaaki Masuda, Masaru Morita, Yasushi Toh, Yoshihiko Maehara, Yutaka Suzuki, Koshi Mimori

**Affiliations:** 1 Department of Surgery, Kyushu University Beppu Hospital, 4546 Tsurumihara, Beppu, 874-0838 Japan; 2 Department of Hepatobiliary-Pancreatic Surgery, National Hospital Organization Kyushu Cancer Center, 3-1-1 Notame, Minami-ku, Fukuoka, 811-1395 Japan; 3 Human Genome Center, Institute of Medical Science, The University of Tokyo, 4-6-1 Shirokanedai, Minato-ku, Tokyo, 108-8639 Japan; 4 Department of Surgery and Science, Graduate School of Medicine, Kyushu University, 3-1-1 Maidashi, Higashi-ku, Fukuoka 813-8582 Japan; 5 Department of Medical Genome Sciences, The University of Tokyo, 5-1-5 Kashiwanoha, Kashiwa, Chiba 277-8562 Japan

**Keywords:** Surgical oncology, Colorectal cancer

## Abstract

**Background:**

We aim to investigate the utility of serial gene mutation tracking for locally advanced CRC in those who underwent curative resection following neoadjuvant chemotherapy.

**Methods:**

We prospectively collected 10 locally advanced CRC cases for which curative resection was performed following preoperative neoadjuvant chemotherapy. Tissues from the primary tumour, distant metastatic tumours, and blood plasma were obtained during serial treatment. Comprehensive mutation analysis of 47 cancer-associated genes was performed using a pre-designed gene panel and next-generation sequencing.

**Results:**

All cases showed a partial response to neoadjuvant chemotherapy, and pathological R0 resection was accomplished. In primary tumours, non-synonymous mutations were detected at between 1 and 14 sites before chemotherapy and at between 1 and 2 sites after. Founder mutations were precisely detected in blood plasma and metastatic tumours during longitudinal treatment.

**Conclusions:**

Serial mutational analysis indicated that subclonal selection occurs during chemotherapy and that plasma can substitute for tumourous tissue in mutational analysis for drug selection and treatment decisions.

## Introduction

Surgical resection is the only curative treatment for CRC, but locally advanced cancer and distant metastasis prevent CRC patients from undergoing curative resection. Considering the high response rate of cytotoxic and molecular-targeted agents for metastatic CRC,^1,2^ neoadjuvant chemotherapy (NAC) designed to down-stage initially non-resectable or marginally resectable locally advanced CRC could allow curative resection and bring a significant survival benefit to patients.^3^ However, there is no standard treatment protocol for locally advanced CRC because systematic studies have not yet been conducted.^4,5^

It is estimated that there are 138 driver genes of CRC, however, one case usually contains only 2–8 driver gene alterations, with the remainder being “passenger” gene defects which have no effect on the neoplastic process.^6,7^ Activating mutations in the *RAS* gene family are well-established biomarkers for a lack of response to anti-epidermal growth factor receptor antibody therapy.^8–10^ Several new biomarkers such as *BRAF* V600E mutation, microsatellite instability, *HER2/neu* overexpression, and *ERBB2* amplification are under investigation for targeted therapies.^11^ We previously revealed the clonal evolution and intra-tumour heterogeneity of CRC with comprehensive exome sequencing and multiregional analysis.^12^ Understanding intra-tumour heterogeneity is clinically important for NAC because it could cause therapeutic failure by fostering evolutionary adaptation.^13^ Although good responses are obtained in patients who receive cytotoxic and molecular targeted therapy for metastatic CRC, most tumours will ultimately progress.^10^ This progression may be attributed to continued clonal evolution and diversification, including resistance to therapy resulting from selective pressure.^14^ Furthermore, the mutational profile from the primary tumour tissue at one time point may not represent the true genomic alteration in the patient, thus it is necessary to identify mutations during serial treatment to maximise the effect of NAC.

To explore and conquer these issues, we obtained tumour and plasma samples from 10 patients with locally advanced CRC. They were acquired consecutively within a prospective tissue collection protocol both at diagnosis and at surgery following oxaliplatin-containing NAC. We tracked serial genomic evolution in locally advanced CRC during disease evolution and following cytotoxic and targeted therapy.

## Materials and methods

### Patients and sample collection

Ten CRC patients with locally advanced primary tumours were recruited into this study. Their primary tumours invaded adjacent organs or tissues and were diagnosed as T4a or T4b using the TNM classification system of the Union for International Cancer Control (UICC). All patients underwent primary tumour resection following oxaliplatin-based NAC at Kyushu University Beppu Hospital. Samples were obtained after written informed consent. The study was conducted in accordance with the Declaration of Helsinki and approved by the Institutional Ethical Review Board of Kyushu University. Detailed clinical characteristics and the sampling protocol are provided in Table S1 and Fig. S1. Tissues were obtained from 3 to 5 locations in the primary tumour by endoscopic biopsy prior to NAC, and DNA was extracted from these samples combined. After NAC, cancer tissues were obtained from surgically resected specimens. In two cases with distant metastatic tumours, tissues were obtained from resected specimens. In two cases that agreed the research with informed consent, 10 mL blood samples were obtained in EDTA tubes and centrifuged to collect the plasma at the time of surgery.

### DNA extraction

Genomic DNA was extracted from formalin-fixed, paraffin-embedded (FFPE) tissue, fresh frozen tumour, adjacent normal intestinal mucosa, and plasma using the QIAamp DNA FFPE Tissue Kit (Qiagen, Hilden, Germany), the AllPrep DNA/RNA Mini Kit (Qiagen), or the QIAamp Circulating Nucleic Acid Kit (Qiagen) as described elsewhere.^15^

### Identification of genomic mutations by next-generation sequencing

Target-enriched libraries for 47 cancer-associated genes were prepared using the ClearSeq Cancer Panel (Agilent Technologies) according to the manufacturer’s instructions (Table S2). Genomic DNA was digested using restriction enzymes, the HaloPlex probe library (Agilent Technologies) was hybridised in the presence of the indexing primer cassette, which resulted in genomic DNA fragment circularisation. The HaloPlex probes were biotinylated, so target DNA-probe hybrids were captured using streptavidin-coated magnetic beads. Next, the target DNA-probe hybrids were ligated closed and PCR amplified to produce a target-enriched library for targeted deep sequencing. The target-enriched libraries were subjected to high-throughput sequencing using a Hiseq 2500 (Illumina, San Diego, CA, USA), and sequencing reads were aligned to the NCBI Human Reference Genome Build 37 hg19 with BWA version 0.7.10 using default parameters (http://bio-bwa.sourceforge.net/). Read depth of the sequencing was provided in Table S3, and mean coverage was between 1284 and 28021. The sequence data were processed using an in-house pipeline (http://genomon.hgc.jp/exome/). The sequencing reads were aligned to the NCBI Human Reference Genome Build 37 hg19 with BWA version 0.5.10 using default parameters (http://bio-bwa.sourceforge.net/). Mutation calling was conducted using the following parameters: (i) mapping quality score ≥ 25, (ii) base quality score ≥ 15, (iii) mismatched bases ≤ 5, (iv) both tumour and normal depths ≥ 100, (v) variant allele frequencies in tumour samples ≥ 0.001 (tissue) or 0.00 (plasma), (vi) Fisher’s exact test *P*-values < 0.05.^12,15,16^ After mutation calling, the Integrative Genomics Viewer (IGV) (http://software.broadinstitute.org/software/igv/) was used to visualise the mutations identified with next-generation sequencing (NGS).^17^

## Results

### Clinical response of locally advanced CRC to NAC

**Fig. 1:**
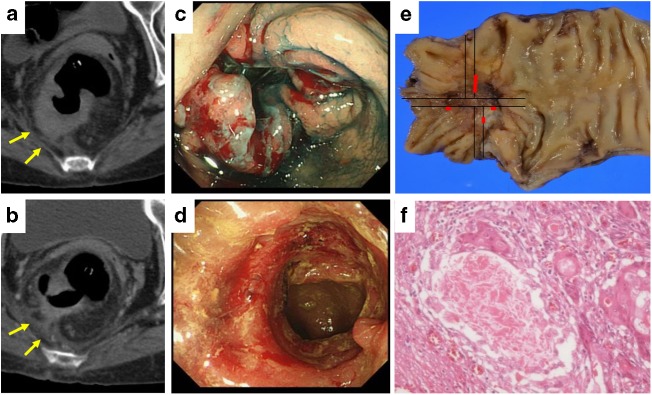
Treatment response of primary tumour to preoperative neoadjuvant chemotherapy. **a**, **b** Computed tomography of primary tumour. The tumour had invaded adjacent retroperitoneum at diagnosis (**a**), and significant shrinkage and fibrosis was seen after chemotherapy (**b**). **c**, **d** Endoscopic findings of the primary tumour. The tumour occupied the whole lumen of the rectum (**c**), and significant response was seen after chemotherapy (**d**). **e** Resected primary tumour specimen. The viable cancer cells are indicated with red lines. **f** Pathological findings of the primary tumour after chemotherapy. More than 2/3 was replaced by fibrosis

All 10 patients showed a partial response in computed tomography and endoscopic colonoscopy according to the revised Response Evaluation Criteria in Solid Tumours (RECIST) guidelines (Fig. 1a–d) ^18^ and underwent complete primary tumour resection without either macroscopic or microscopic residual tumour. The pathological response of the primary tumour was evaluated using the percent of the area showing fibrosis and necrosis (Fig. 1e, f). Five cases (50%) showed >66% tumour regression (grade 2), 3 cases (30%) showed 34–66% regression (grade 1b), and 2 cases (20%) showed <33% regression (grade 1a) according to pathological findings.

### Gene mutational tracking during preoperative chemotherapy

Mutations were identified in between 1 and 14 genes in primary tumour obtained by endoscopic biopsy before treatment (Table 1). A mutation in *TP53* was identified in 9 of the 10 cases (90%), and a mutation in *KRAS* or *NRAS* was identified in 5 cases (50%). After NAC, the number of mutations decreased, and only 1 or 2 gene mutations, which were *RAS* and *TP53* mutations exclusively found in the primary tumour biopsy before NAC, were detected in the primary tumours (Table 1). In 1 case, mutations in *KRAS* and *PTEN* were detected before chemotherapy, but no mutations were detected after chemotherapy. In 2 metastatic tumours in the liver and spleen, the same mutations were found as in the resected primary tumours (Table 1).

**Table 1:** Genes identified nonsynonymous mutations by serial mutational analysis of locally advanced colorectal cancer during longitudinal treatment

Case no.	Primary tumour biopsy (prechemotherapy) gene name (VAF)	Primary tumour resection (postchemotherapy) gene name (VAF)	Plasma (postchemotherapy) gene name (VAF)	Metastatic tumour gene name (VAF)
1	*TP53* ^*S109F*^ *(0.249), FGFR3* ^*P250R*^ *(0.028), NOTCH1* ^*E1567X*^ *(0.056), ERBB2* ^*R849Q*^ *(0.062)*	*TP53* ^*S109F*^ *(0.550)*	no data	no data
2	*TP53* ^*G113S*^ *(0.174), ERBB2* ^*G776V*^ *(0.117), ERBB4* ^*R938H*^ *(0.060), FGFR3* ^*P250R*^ *(0.019), EGFR* ^*V845M*^ *(0.117), RUNX1* ^*A384S*^ *(0.138), FANCA* ^*E950X*^ *(0.083)*	*TP53* ^*G113S*^ *(0.013)*	no data	*TP53* ^*G113S*^ *(0.621)*
3	*KRAS(0.200)*,^*Q61L*^ *PTEN*^*E288fs*^	no mutation	no data	no data
4	*KRAS* ^*G12D*^ *(0.337), TP53* ^*H61L*^ *(0.113)*	*KRAS* ^*G12D*^ *(0.319), TP53* ^*H61L*^ *(0.526)*	*KRAS* ^*G12D*^ *(0.003), TP53* ^*H61L*^ *(0.002)*	no data
5	*KRAS*^*G13D*^*(0.421), TP53*,^*105_108del*^ *FGFR3*^*A265E*^*(0.079), MAP2K4*^*D276Y*^*(0.078)*	*KRAS* ^*G13D*^ *(0.780), TP53* ^*105_108del*^	no data	*KRAS* ^*G13D*^ *(0.463), TP53* ^*105_108del*^
6	*TP53* ^*R43H*^ *(0.202)*	*TP53* ^*R43H*^ *(0.412)*	*TP53* ^*R43H*^ *(0.022)*	no data
7	*TP53* ^*P59P*^ *(0.052), FGFR3* ^*T264M*^ *(0.024)*	*TP53* ^*P59P*^ *(0.052)*	no data	no data
8	*KRAS* ^*G12V*^ *(0.387), TP53* ^*R116Q*^ *(0.047), RET* ^*P766L*^ *(0.040), SMAD4* ^*R445X*^ *(0.033), STK11* ^*E256K*^ *(0.027), ALK* ^*R1275X*^ *(0.022), RUNX1* ^*R180Q*^ *(0.026), PIK3CA* ^*N345K*^ *(0.165), FGFR3* ^*A391V*^ *(0.028), MET* ^*E168D*^ *(0.113), CDKN2A* ^*R71C*^ *(0.053), FANCG* ^*D437H*^ *(0.028), ABL1* ^*D276E*^ *(0.048), NOTCH1* ^*R1598R*^ *(0.056)*	*TP53* ^*R116Q*^ *(0.096), MET* ^*E168D*^ *(0.352)*	no data	no data
9	*NRAS* ^*Q61L*^ *(0.219), TP53* ^*R116Q*^ *(0.030), MAP2K4* ^*I113T*^ *(0.256)*	*NRAS* ^*Q61L*^ *(0.200), TP53* ^*R116Q*^ *(0.021)*	no data	no data
10	*TP53* ^*C110F*^ *(0.280), PIK3R1* ^*L340L*^ *(0.038)*	*TP53* ^*C110F*^ *(0.395)*	no data	no data

*VAF* variant allele frequency

### Tracking mutations in circulating tumour DNA during NAC

**Fig. 2:**
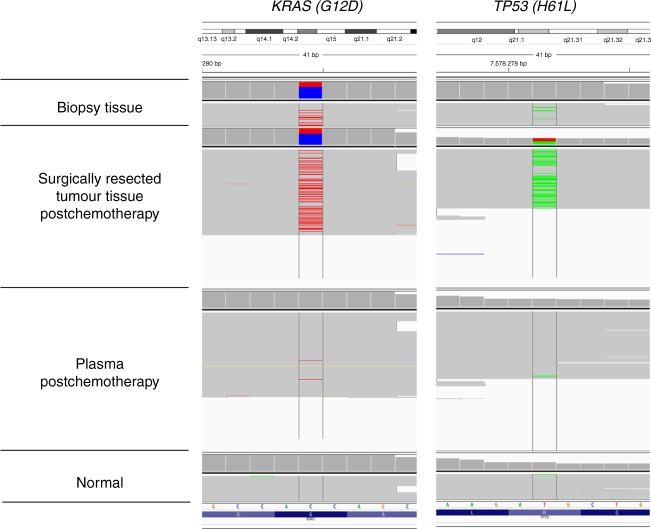
Visualization of gene mutations in primary tumour and plasma with Integrative Genomics Viewer. A representative mutation in the primary tumour at diagnosis, in the surgically resected tumour, in plasma, and in normal samples. Nonsynonymous mutations in *KRAS* (chr12_25398284) and *TP53* (chr17_7578271) are visualized

Serial mutational analysis may be necessary in patients with conversion surgery following NAC because various genomic modifications could result from chemotherapy. We explored mutant circulating tumour DNA (ctDNA) in blood samples after NAC in two patients. In one case, mutations in *KRAS* and *TP53* were detected from a biopsy specimen before chemotherapy. After 4 courses of CAPOX therapy, the same mutations were detected in ctDNA (Fig. 2). Those mutations were also verified from surgically resected tissues after NAC. In another case, a driver mutation in *TP53* coincident with the primary tumour tissue was also detected in ctDNA. These results indicate that plasma can be used for repeated and noninvasive serial monitoring of mutational changes.

## Discussion

This is the first clinical series showing serial gene mutation tracking in patients with locally advanced CRC. The tumours responded well to NAC, and all patients eventually underwent pathologically complete resection. Our results indicate that down-staging by NAC could be a standard strategy for treating locally advanced CRC. It is important to maximise the anti-tumour effect with the optimal regimen and duration of chemotherapy, otherwise it is possible that the patients will miss their chance for surgical resection because of an adverse event or tumour progression during preoperative chemotherapy. Serial tracking of gene mutations can be a useful biomarker for preoperative chemotherapy because it can monitor treatment response, tumour burden, and resistant clones.^19,20^

In this study, the number of mutations decreased after NAC. Most founder mutations such as *KRAS* and *TP53* remained throughout chemotherapy, but other passenger mutations were not found after exposure to cytotoxic and molecular-targeted agents. These results suggest that the certain subclones are diminished by a massive response to NAC. In one case, mutations found in the primary tumour at diagnosis were not detected after NAC. In this case, massive fibrosis and necrosis were found in the pathological analysis, suggesting the strong selection of subclones or small content of cancer cells from neoadjuvant treatment may cause changes in the mutational phenotype. Our data indicate that serial tracking of gene mutations could detect dynamic genomic evolution during chemotherapy maximise the anti-tumour effects of molecular profile-based precision medicine.

The source of ctDNA appears to be mainly cell death, thus ctDNA from tumours in blood has been reported as a biomarker for early detection, tumour burden, and response to treatment.^21–27^ We found founder mutations consistent with the primary tumour in plasma samples after chemotherapy, indicating that ctDNA could be used instead of tissue samples as a ‘liquid biopsy’ to detect tumour-specific mutations for better patient selection and treatment individualisation throughout multiple lines of therapy.^28^ Using ctDNA has advantages for the serial monitoring of genetic and epigenetic alterations because samples can be obtained noninvasively and repeatedly. The other issue to be highlighted regarding ctDNA is that it could be beneficial to evaluate molecular profiles beyond intra- and inter-tumour (primary and metastatic, etc.) molecular heterogeneity.^12,29^ We analysed a post-NAC plasma, but the serial mutational tracking of pre- and post-NAC plasma would be warranted for further studies.

The main limitation of this study is the small patient cohort size. Although this is a preliminary study, to our knowledge this is the first published data of serial mutational tracking during NAC and surgery for locally advanced CRC. Our serial mutational analysis illustrates that dynamic mutational changes occur during subclonal NAC for locally advanced CRC. These findings show the utility of mutational tracking for drug selection and treatment decisions.

## Electronic supplementary material

Supplementary Figure1

Supplementary Table1

Supplementary Table2

Supplementary Table3

Supplementary Figure S1 legend
