# Supplementary figures and images for: Serial mutational tracking in surgically resected locally advanced colorectal cancer with neoadjuvant chemotherapy
Source: Br J Cancer. 2018 Aug 3;119(4):419–23. doi: 10.1038/s41416-018-0208-5 (PMC6134007; doi:10.1038/s41416-018-0208-5)

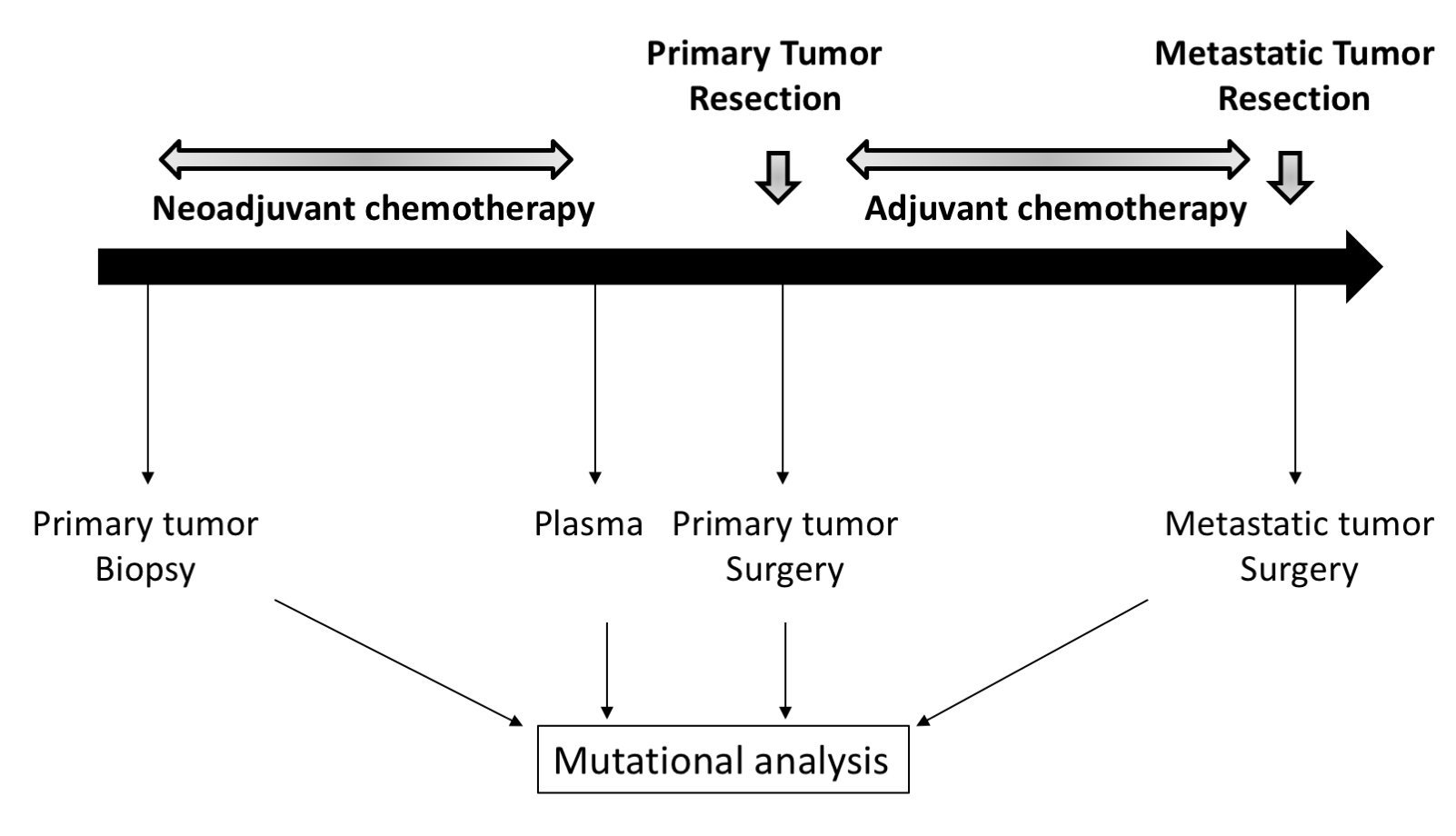

Supplement: Supplementary file 1 — Supplementary Figure1 [file 41416_2018_208_MOESM1_ESM.tif]
