# Supplementary material for: Serial mutational tracking in surgically resected locally advanced colorectal cancer with neoadjuvant chemotherapy
Source: Br J Cancer. 2018 Aug 3;119(4):419–23. doi: 10.1038/s41416-018-0208-5 (PMC6134007; doi:10.1038/s41416-018-0208-5)
Supplement: Supplementary file 5 — Supplementary Figure S1 legend [file 41416_2018_208_MOESM5_ESM.docx]

**Fig. S1. Overview of study treatment course and sampling.**

Primary tumour tissue was collected before neoadjuvant chemotherapy. Plasma and primary tumour tissue were collected at the time of surgery. In 2 cases, tissues from metastatic tumours were collected when surgery was performed.
